# Paying for Sex Among Heterosexual Men in Israel: The Role of Gender Role Conflict, Distress, and Attitudes

**DOI:** 10.1007/s10508-025-03262-8

**Published:** 2025-11-05

**Authors:** Guy Shilo, Inbar Malka, Einat Peled

**Affiliations:** School of Social Work, Tel-Aviv University, Tel-Aviv, 6139001 Israel

**Keywords:** Men who pay for sex, Gender role conflict, Attitudes toward paying for sex, Israel, Distress

## Abstract

Diverse perceptions of paying for sex notwithstanding, it is acknowledged as a societal concern in many countries worldwide, with no consensus reached regarding the most appropriate policies. The present study directed its attention toward heterosexual men who pay for sex and sought to explore the potential influences of distress and gender role conflict (GRC) on the likelihood of sex payment, while taking into consideration attitudes toward paying for sex as a mediating factor. Data were collected from a quota sample of 934 heterosexual Israeli men. Of all sociodemographic variables, regression analysis showed that lower level of religiosity was the only significant predictor of sex payment. Above and beyond religiosity, higher level of distress, the GRC scales of Restrictive Emotionality, Success/Power/Competition, and Conflict Between Work and Family Relations, and attitudes of paying for sex as a legitimate behavior were significant predictors of sex payment. Conversely, lower levels of Restrictive Affectionate Behavior Between Men and attitudes viewing paying for sex as a deviant behavior were also found to be significant predictors of paying for sex. Moreover, attitudes toward paying for sex fully mediated the association between distress, most of the components of GRC, and paying for sex. Our findings highlight the interconnectedness between masculine role socialization and men’s psychological experiences, and the potential relevance of these associations to men’s involvement in sex payment. Furthermore, the significant role of attitudes toward paying for sex in the actualization of sex payment is discussed.

## Introduction

There is currently no global consensus regarding how to address the phenomenon of paying for sex (Benoit et al., 2019). Prominent international health and human rights organizations, including the World Health Organization (2020), Human Rights Watch (2019), and the Council of Europe (2024), oppose the criminalization of both women who are paid for sex and men who pay for sex, and instead call for policies that reduce stigma and support the dignity, safety, and mental well-being of individuals engaged in the sex industry. At the same time, a growing number of Western countries, including Israel, where this study was conducted, have attempted to address paying for sex in recent years through the implementation of "End Demand" legislation. This legislative approach entails the criminalization of sex payment, while concurrently providing educational programs for individual who pay for sex, mostly men (Shamir et al., 2023).

Several qualitative studies have highlighted the role of masculine identity in the context of paying for sex, as well as the relationship between psychological distress and this behavior (see Prior & Peled, 2021, qualitative meta-synthesis). However, these associations have been largely overlooked in quantitative research, despite their potential contribution to a deeper understanding of the psychological dimensions of sex payment and the development of informed policy. Furthermore, international health and human rights organizations call to promote mental well-being among individuals involved in the sex industry. Thus, the present study focuses on men who pay for sex (MPS), aiming to examine how distress and gender role conflict may be associated with the likelihood of engaging in sex payment.

### Paying for Sex and Gender Role Conflict

MPS report discomfort that often extends beyond individual psychological struggles and reflects broader societal pressures and gendered expectations. Many MPS describe experiencing significant anxiety related to normative ideals of masculinity, particularly around sexual performance, the value placed on men sexual experience, and fears of vulnerability in intimate interactions with women. In this context, paying for sex may serve as a coping mechanism that offers a controlled and less threatening environment in which men can confront or avoid these insecurities (Huysamen, 2020). Thus, paying for sex can be seen not only as a personal choice but also as a socially shaped response to rigid and often unattainable gender norms. This pattern of behavior is closely linked to the broader construct of gender role conflict, which captures the psychological strain men experience when they perceive themselves as failing to meet culturally prescribed standards of masculinity.

Gender role conflict (GRC) is a theoretical, research-based concept illustrating how socialization to rigid gender roles can limit an individual’s potential. Men may experience varying degrees of this restriction due to societal norms learned in sexist and patriarchal societies. GRC can have a detrimental effect on multiple aspects of men’s lives, leading to anxiety, depression, addiction, and even suicide (O’Neil, 2008). The conceptualization of GRC has gained significant research interest (O’Neil, 2008, 2013). It posits four behavioral patterns that depict masculine expectations: (1) Success/ Power/ Competition (SPC)—personal attitudes toward success that are pursued through competition and power; (2) Restrictive Emotionality (RE)—limitations in expressing and articulating one’s emotions; (3) Restrictive Affectionate Behavior Between Men (RABBM)constraints on expressing one’s thoughts and feelings to other men, as well as engaging in physical touch with other men; and (4) Conflict Between Work and Family Relations (CBWFR)—impediments to achieving a balance between work, school, and family relations, resulting in health problems, overwork, stress, and a dearth of leisure and relaxation (O’Neil, 2008; O’Neil et al., 1986). As expanded below, each of these four patterns relates to paying for sex.

### Paying for Sex and Success/Power/Competition

The literature suggests that MPS construct and perform their masculinity and sexuality through their experiences of paying for sex. As Prior and Peled’s (2021) qualitative meta-synthesis elucidates, the construction of identity among men who pay women for sex is heavily influenced by the predominant social discourse of masculinity. Paying for sex is often understood as alleviating identity conflicts among men, as it facilitates the attainment of societal standards pertaining to masculinity, sexuality and power (Prior & Peled, 2021). Thus, there are grounds to assume that paying for sex is associated with the SPC factor of GRC.

A paramount characteristic of successful hegemonic masculinity is the possession of sexual desirability, experience, and proficiency (Barker & Ricardo, 2005). Men are suggested to embody and perform such idealized, successful (hetero) sexuality within their interaction with women who are paid for sex (Huysamen & Boonzaier, 2015). For instance, participants in Huysamen’s (2020) study described sex payment as allowing them to experience successful sexual masculinity through losing virginity and creating optimal conditions to potent sexual performance with no possible rejection. In her study among Israeli MPS, Lahav-Raz (2020) described how paying for sex serves as a platform for the enactment of power relations among the men through ‘hunting’ women on the streets.

Indeed, it has been long argued that paying for sex represents a manifestation of masculine dominance, power and control over women (Dworkin, 1981; Jeffreys, 1997; MacKinnon, 1987, 1989). Prior and Peled’s (2021) review suggests that paying for sex establishes power-related identity constructions within the men’s interactions with women who are paid for sex. For instance, a qualitative study conducted by Joseph and Black (2012) found that some men pay women for sex because they feel rejected by women, unattractive to them, and lack confidence in relating to them. Interestingly, another study found a link between paying for sex and subordination to women (Closson et al., 2020). Similarly, Huysamen’s (2020) study demonstrated that MPS deal with their feelings of humiliation and powerlessness by humiliating and dominating women who are paid for sex. According to a systematic review by Wilcox et al. (2009), certain men are indeed motivated to engage in sex payment as a means of exerting control over another individual, and bolstering their sense of potency (Månsson, 2006).

### Paying for Sex and Restrictive Emotionality

Restrictive emotionality is another aspect of hegemonic masculinity (Birch et al., 2017) that seems to be associated with sex payment (Closson et al., 2020). Overall, studies suggest that emotionality plays a crucial role in sex payment (Huysamen, 2019). For instance, some MPS present considerable ambivalence toward female sexuality, which they perceive as manipulative and overly emotional (Huysamen & Boonzaier, 2015). MPS in Tal-Hadar et al. (2022) study indeed report difficulties with emotional intimate attachment and a romantic history of mainly short relationships. Similarly, Hammond and van Hooff (2020) contend that MPS opt for sex payment as a rational choice to contain their emotions. Others found that emotionless sex with no commitment or attachment to women is one of the motivations mentioned for engaging in paying for sex (Hammond & van Hooff, 2020; Wilcox et al., 2009).

Simultaneously, the emotional intimate needs of MPS are widely acknowledged. Paying for sex provides some men with a ‘girlfriend experience’, which involves an encounter of affection, intimacy, emotional reciprocity, and friendship (Bernstein, 2007; Prior & Peled, 2021; Wilcox et al., 2009). Further, paying for sex was perceived to fulfill a spectrum of men’s sexual and intimate needs without compromising their masculine self-image (Tal-Hadar et al., 2022). For certain men, sex payment serves as a source of emotional connection and in some cases, constitutes a primary emotional relationship (Levi, 2020; Sanders, 2006, 2008) or evolves to a meaningful relationship (Milrod & Weitzer, 2012).

### Paying for Sex, Restrictive Affectionate Behavior Between Men, and Conflict Between Work and Family Relations

GRC restricts men’s satisfaction with relationships with family members and friends and it has been reported that men who prioritize family values report higher levels of CBWFR (Leka, 1998; O’Neil, 2008). Similar difficulties were reported by MPS. For instance, men who engage in transactional sex frequently defined ideal masculinity as working hard, meeting one’s wife’s needs and raising the children well, and an overall duty to meet expectations at home (Wentzell, 2014). Overall, it is reasonable to assume that the difficulties MPS frequently report with intimacy and commitment, as previously illustrated, impair relationships in their lives, thus indicating a possible association between paying for sex and CBWFR.

Difficulties of MPS with relationships and emotionality are related also to the GRC factor of RABBM. There is a lack of direct research on the relationships of MPS with men. However, we suggest that it is reasonable to assume a correlation between paying for sex and RABBM because sex payment is frequently viewed as a means to conform to the norms of hegemonic masculinity (e.g., Shumka et al., 2017), and because hegemonic masculinity is strongly associated with compulsory homophobia (Haywood & Mac an Ghaill, 2012) and relationships between men that are low in expressiveness and high in instrumentality, restraint, and physical and mental toughness (Migliaccio, 2009).

### Paying for Sex and Distress

The literature about MPS suggest that paying for sex can either stem from emotional distress or cause significant distress, as stated by the men themselves, by professionals who work with them and by women who are paid for sex, and will be demonstrated below. The roots of this distress can vary between MPS and the distress can manifest through various emotions.

Distress can be the primary motivation for sex payment. A study among 597 MPS has reported that for some (5.4%) stress is stated as the reason they first initiated paying for sex (Kennedy et al., 2004). Paying for sex is indeed described by men as providing relief of the many stressful responsibilities of life (Plumridge et al., 1997). However, the distress experienced by some MPS may have deeper roots. For instance, loneliness is a significant motivation for sex payment (Kennedy et al., 2004). Mansson’s (2006) study suggests that MPS who have potency and control vulnerabilities due to looks, physical or mental disabilities, seek women who are paid for sex to fulfill relationship needs. Some of the men in Huysamen’s (2020) study expected women who are paid for sex to be a container of their anxieties and help them with problems related to sexuality and self-esteem. Others tie paying for sex to men’s reluctance to commit to a long-term conventional relationships or to difficulties with current intimate relationships (Huysamen, 2020; Wilcox et al., 2009), which can both reasonably involve some distress.

Women who are paid for sex seem to support the notion that paying for sex serves to alleviate distress for some men. Sex workers in Sanders’ (2006) study reported that MPS often shared with them personal issues and secrets, including depression and even suicidal thoughts, and viewed their work as resembling that of mental health professionals.

Studies among help providers also suggest distress as a potential factor leading to paying for sex. In a recent study conducted in Sweden, social workers described their clients’ motivations to pay for sex as involving anxiety, depression, addiction, trauma or overall psychological ill-health (Grönvall, 2023). Similarly, Israeli therapists in Ben-Shalom Fishbein’s (2020) study linked paying for sex to psychological distress stemming from disruptive formations of object relations. Lemma (2015) presented similar analytic notion, according to which paying for sex is men’s way to restore mental representations of the self and others (Ben-Shalom Fishbein, 2020).

Distress in MPS can be also understood in the context of sex addiction (e.g., Gordon-Lamoureux, 2007) and there is a substantial comorbidity of paying for sex with substance use disorders or other behavioral addictions like gambling (Carnes et al., 2005; Hartman et al., 2012; Stavro et al., 2013). In that sense, paying for sex is seen as a manifestation of sex addiction or multiple addictions, involving a wide range of detrimental outcomes, significant distress and feelings of shame and guilt (Carnes et al., 2005; Garcia & Thibaut, 2010; Watson & Vidal, 2011).

Engaging in sex payment can also cause distress in certain men, as observed by Watson and Vidal (2011) who treated MPS. They noticed that their patients were highly concerned or distressed about their behavior and for many, paying for sex provoked an internal conflict. MPS may simply fear that their behavior will be exposed within their current intimate relationship, or feel self-denigration due to internalizing critical social perceptions of paying for sex as a sickness and pervertion (Lahav-Raz et al., 2023). According to Weitzer’s (2009) review, paying for sex can indeed provoke negative feelings of fear, shame and guilt. For instance, Levi (2020) found such feelings among Israeli Jewish religious and Orthodox MPS. Other study’s demonstrated how paying for sex can be an overall agonizing and diminishing experience for men, even when they did it in order to ease emotional problems (Prior & Peled, 2025a, 2025b; Tal-Hadar et al., 2022).

Occasionally, as reported both by therapists who treat MPS and by the men themselves, some men experience distress due to a moral conflict relating to the suffering of the women who are paid for sex or the exploitative nature of the sex industry, wish to cease paying for sex but are unable to do so (Ben-Shalom Fishbein, 2020; Lahav-Raz, 2022; Prior & Peled, 2025a, 2025b).

### Attitudes Toward Paying for Sex

Attitudes toward paying for sex and toward MPS vary between countries and populations, and are largely divided by two competing views—regarding it as normative or deviant (Cotton et al., 2002; Jonsson & Jakobsson, 2017; Peled et al., 2021, Shamir et al., 2023). Not surprisingly, research shows that MPS hold favorable attitudes toward paying for sex. Both Farley (2011) and Durchslag and Goswami (2008) found that men who engage in sex payment are more inclined to perceive paying for sex as a normal behavior for men.

More importantly, attitudes of MPS toward paying for sex can play a crucial role in the frequency of sex payment. Peled et al., (2021) found low levels of perceiving paying for sex as a legitimate, acceptable behavior and the right of unfortunate men among men who never paid for sex, medium levels in men who paid only once and high levels in repeated MPS. Moreover, there where high levels of perceiving paying for sex as a deviant, immoral and exploitative behavior in men who never paid and in men who paid only once, compared to low levels in repeated MPS. These findings demonstrate how attitudes toward paying for sex predict the frequency of sex payment among men, with relatively negative attitudes decreasing the frequency of sex payment even for MPS. The authors concluded that the attitudes of men who paid for sex once may indicate a moral conflict regarding paying for sex, that may prevent men from repeating the behavior (Peled et al., 2021).

Therefore, we propose that attitudes toward paying for sex may potentially play a significant role in engaging in sex payment. We suggest that while distress and GRC may be present, they alone do not suffice to predict paying for sex. It is the attitudes toward this behavior that may ultimately serve as a catalyst in the actualization of sex payment. To the best of our knowledge, to date only one study has delved into the correlation of attitudes toward paying for sex with GRC (but not with distress), and found that men with higher GRC tend to exhibit normative attitudes toward paying for sex and women who are paid for sex, but deviant attitudes toward MPS (Radosh Sverdlin, 2020)—illustrating an association between GRC and attitudes toward paying for sex.

## Summary and Hypotheses

Based on the literature review above, which indicates that distress serves as both an antecedent and a result of paying for sex, and describe how different aspects of paying for sex align with prevailing masculine culture and consequently have the potential to alleviate gender role conflict, we hypothesized that:

1. Higher levels of distress and GRC among men will be associated with paying for sex.
2. Attitudes toward the behavior will mediate the associations between distress and GRC and paying for sex.

While previous research primarily approached this topic through qualitative methods or indirectly, our study is the first attempt at a quantitative exploration of these associations.

## Method

### Participants and Procedure

The sample for this study was obtained through an online survey conducted in January 2020, using a research panel consisting of 80,000 Israeli panelists. A total of 2,012 adults participated in the survey (Mohajan, 2020). The survey used a quota sample, designed to represent the characteristics of age, gender, place of residence, family status, and level of religiosity of the Jewish-Israeli adult population (Israeli Central Bureau of Statistics, 2018; see more details in Shamir et al., 2023). Potential participants were contacted by the company and invited to complete a 20 min online questionnaire. Those who consented to participate filled out the survey until the required sample quota—based on predefined demographic criteria—was met. For this study, the sub-sample of 934 men who self-identified as heterosexual was used (age: *M* = 42.6, *SD* = 15.37).

### Measures

Attitudes toward men who pay for sex were assessed using the Attitudes Toward Men Who Pay for Sex Scale (Shilo et al., 2020). The 32 item measure is comprised of two subscales: Paying for sex as a legitimate behavior (19 items, e.g., “going to a prostitute is an acceptable manly behavior”; “buying sex services is like buying any other service”), and Paying for sex as a deviant behavior (13 items, e.g., “men who go to prostitutes are immoral”; “many men who go to prostitutes are the criminal type”).^1^ Respondents were required to endorse their agreement with each item on a scale ranging from 1 (*strongly disagree*) to 5 (*strongly agree*). In this study, internal consistency for the Paying for sex as a legitimate behavior subscale was α = .90, and for the Paying for sex as a deviant behavior subscale, *α* = .88. Scores were calculated as the mean of each of the subscale items; the higher the score, the greater the agreement with the view that paying for sex is a legitimate or deviant masculine behavior, respectively.

Mental distress was assessed by means of the distress scale in the Mental Health Inventory (MHI; Veit & Ware, 1983), using the Hebrew short version (Izack, 2002) that contains 8 items. This is a widely used measure of psychological distress and well-being that provides a global mental health index. The distress scale consists of 8 items that gauge anxiety, depression, and loss of control (e.g., “How much time in total have you felt depressed in the past month?”). Items are administered on a 5-point scale ranging from 1 (Never) to 5 (Always). Scores were calculated as the mean score of the items: the higher the score, the greater the distress. In the current study, the Cronbach’s *a* was 0.91.

Gender Role Conflict was measured using the Gender Role Conflict Scale—Short Form (GRC-SF). The 16-item scale is a measure of men’s reactions to the inconsistent and unrealistic gender role expectations they face and the negative psychological state that results from these. The GRC-SF, developed by Wester (2012), is based on O’Neil et al.'s (1986) original GRC scale. The scale is divided into four subscales, each addressing a different dimension of GRC represented by four items. Success, power, and competition (SPC; e.g., “Winning is a measure of my value and personal worth”; “I strive to be more successful than others”); Restrictive emotionality (RE; e.g., “Talking about my feelings during sexual relations is difficult for me”; “I have difficulty expressing my tender feelings”); Restrictive affectionate behavior between men (RABBM; e.g., “Men who touch other men make me uncomfortable”; “Being very personal with other men makes me feel uncomfortable”); Conflict between work and family relations (CBWFR; e.g., “Finding time to relax is difficult for me”; “My needs to work or study keep me from my family or leisure more than I would like”. Participants rate each of the 16 items on a 6-point Likert scale, ranging from 1 (*strongly disagree*) to 6 (*strongly agree*). scores for each subscale that is obtained by the mean of each sub-scales’ four items. The higher the score the more Restrictive emotionality (*α* = .80); Success, power, and competition (*α* = .73); Restrictive affectionate behavior between men (*α* = .79); and conflict between work and family relations (*α* = .78) a man has.

Demographics. Participants indicated their age, gender (man, woman, other), sexual orientation (heterosexual, gay/lesbian, bisexual), family status, level of monthly income (on a scale ranging from 0 – “no income” to 5 – “well above average”), level of religiosity (on a scale ranging from 1—“secular” to 4—“Orthodox”). Participants were asked whether they ever paid for sex or used sexual services (even if someone else paid for them), not including pornography (yes/no).

### Data Analysis

First, we examined the relationships between the key study variables and several sociodemographic characteristics. For this purpose, we used Pearson correlations to assess the strength and direction of associations between two continuous variables, and eta correlations to examine associations between continuous variables and binary (yes/no) variables.

Following this preliminary step, we conducted a series of three binary logistic regression analyses to explore which variables were associated with the likelihood of paying for sex (Hypothesis 1). Logistic regression is a statistical technique commonly used to predict the probability of a binary outcome (in this case, whether or not a participant has ever paid for sex), based on one or more predictor variables.

In all three regression models, we first controlled for age, income level, and religiosity—three sociodemographic variables that were found to be significantly related to paying for sex in the initial correlation analyses. These variables were entered into the regression models as covariates in the first step. In the first regression model, we entered the psychological variables—mental distress and the four components of GRC—to test whether they were associated with paying for sex, above and beyond the covariates. In the second model, we examined the role of participants’ attitudes toward paying for sex, specifically whether they viewed it as a legitimate or deviant behavior. These attitude variables were added as a second step in the regression to assess their direct association with paying for sex. In the third model, we included all relevant variables—both psychological and attitudinal—in order to test whether attitudes toward paying for sex helped to explain (i.e., mediate) the relationship between mental distress, GRC, and the likelihood of paying for sex (Hypothesis 2). This model followed the well-established framework for testing mediation outlined by Baron and Kenny (1986).

To further validate the mediation effects, we conducted a bootstrapped mediation analysis using the PROCESS macro for SPSS (Model 4; Hayes, 2012). This approach involves resampling the data 5,000 times to generate bias-corrected confidence intervals for the indirect effects (Preacher & Hayes, 2008). If these confidence intervals (CI) do not include zero, the mediation effect is considered statistically significant. As in the regression models, age, income, and religiosity were included as control variables in these analyses.

Prior to conducting the main analyses, the data were screened for univariate and multivariate outliers, and for multicollinearity. Univariate outliers were examined using standardized z-scores (|z|> 3.29), and multivariate outliers were evaluated based on standardized residuals and leverage values in the regression models. No extreme values were identified that exceeded conventional thresholds or unduly influenced the results. Therefore, all cases were retained for analysis. Multicollinearity diagnostics indicated no concern, with all tolerance values > .66 and all variance inflation factors (VIFs) < 1.51, well below the commonly accepted thresholds of concern (VIF > 5, Tolerance < .20).

## Results

Among all men, 308 (33%) declared that they have paid for sex at least once in their lifetime. Table 1 presents the bivariate analysis. As can be seen, paying for sex was positively and significantly correlated with higher levels of distress, restrictive emotionality, success power and competition, conflict between work, and family relations, and attitudes of paying for sex as a legitimate behavior, and negatively and significantly correlated with restrictive affectionate behavior between men, and attitudes of paying for sex as a deviant behavior. In addition, MPS tended to be older, with lower levels of income and lower levels of religiosity.

**Table 1:** Inter-correlations of study variables and demographic variables (*N* = 934)

	Variable	1	2	3	4	5	6	7	8	9	10
1	Age	1									
2	Level of income	−.04	1								
3	Religiosity	−.23***	−.08**	1							
4	Paying for sex (yes)	.08*	−.08**	−.19***	1						
5	Paying for sex as legitimate behavior	.04	−.05	−.20***	.32***	1					
6	Paying for sex as deviant behavior	−.27***	.01	.32***	−.22***	−.18***	1				
7	Distress	−.15***	−.09**	−.07*	.11***	.24***	.06*	1			
8	RE	−.04	−.01	−.10**	.09**	.22***	.08*	.29***	1		
9	SPC	−.18***	−.01	.08*	.08**	.19***	.08*	.18***	.26***	1	
10	RABBM	.10**	−.06	.10**	−.10**	.11***	.12***	.16***	.43***	.32***	1
11	CBWFR	−.25***	−.04	.07*	.10**	.14***	.14***	.42***	.39***	.24***	.24***

^*^p < .05; **p < .01; ***p < .001; RE = Restrictive Emotionality; SPC = Success, Power and Competition; RABBM = Restrictive Affectionate Behavior Between Men; CBWFR = Conflict Between Work and Family Relations

### Variables Associated with Paying for Sex (Hypothesis 1)

Logistic regression analyses (see Table 2) revealed that among all socio-demographic variables, religiosity was the only significant predictor of paying for sex. Specifically, each unit increase in religiosity was associated with a 38% reduction in the odds of engaging in sex payment. Beyond religiosity, several psychological variables emerged as significant predictors in Model 1, including mental distress, restrictive emotionality (RE), success, power, and competition (SPC), and CBWFR. Each unit increase in these variables was associated with a 19% to 25% increase in the odds of paying for sex. In Model 2, the two attitudinal variables were found to be highly significant: viewing paying for sex as a legitimate behavior was associated with a 145% increase in the odds of having paid for sex, while viewing it as a deviant behavior was linked to a 42% reduction in those odds. Notably, RABBM was also a significant negative predictor, with higher levels associated with a 35% decrease in the odds of paying for sex. Among all variables examined, the attitudes toward paying for sex—both as legitimate and deviant—emerged as the strongest predictors of paying for sex.

**Table 2:** Logistic regression predicting paying for sex (*N* = 934)

	Model 1				Model 2				Model 3			
Predictor	B	SE	OR	95% CI	B	SE	OR	95% CI	B	SE	OR	95% CI
*Step 1*												
Age	.01	.01	1.01	.90–1.01	.01	.01	1.01	.90–1.01	.01	.01	1.01	.90–1.01
Level of income	−.07	.06	.94	.80–1.05	−.07	.06	.94	.80–1.05	−.07	.06	.94	.80–1.05
Religiosity	−.49	.09	.62***	.51–.74	−.49	.09	.62***	.51–.74	−.49	.09	.62***	.51–.74
*Step 2*												
Distress	.07	.10	1.25*	1.02–1.39					.01	.11	1.01	.81–1.24
RE	.14	.08	1.23*	1.07–1.37					.10	.09	1.11	.94–1.31
SPC	.18	.08	1.21*	1.05–1.38					.07	.09	1.05	.91–1.27
RABBM	−.12	.07	.65**	.74-.97					−.11	.08	.89	.77–1.03
CBWFR	.18	.08	1.19*	1.01–1.37					.21	.09	1.24*	1.05–1.47
Paying for sex as a legitimate behavior					.89	.11	2.45***	1.97–3.03	.82	.12	2.27***	1.81–2.85
Paying for sex as a deviant behavior					−.54	.10	.58***	.48–.71	−.59	.11	.55***	.45–.68

^*^p < .05; **p < .01; ***p < .001; RE = Restrictive Emotionality; SPC = Success, Power and Competition; RABBM = Restrictive Affectionate Behavior Between Men; CBWFR = Conflict Between Work and Family Relations; CI = Confidence Interval for Odds Ratio (OR)

### Attitudes Toward Paying for Sex as a Mediator (Hypothesis 2)

As can be seen in model 3 (see Table 2), when adding the mediation variables of attitudes toward paying for sex to the regression equation, the predictions of levels of distress, RE, SPC, and RABBM were no longer significant, suggesting full mediation. Mediation analyses (using bootstrapping methods with bias-corrected confidence estimates; Preacher & Heyes, 2008) confirmed the significance of the indirect effects of the relations between mental distress (CI: .12, .28), RE (CI: .02, .16), SPC (CI: .07, .18), RABBM (CI: .02, .11) and paying for sex, when attitudes toward paying for sex as a legitimate behavior is the mediator; and the significance of the indirect effects of the relations between mental distress (CI: -.09, -.02), RE (CI: -.08, -.01), SPC (CI: -.08, -.01), RABBM (CI: -.08, -.02) and sex payment when attitudes toward paying for sex as a deviant behavior is the mediator. As can be seen in Table 2, the attitudes toward paying for sex variables did not mediate the direct prediction of paying for sex by CBWFR.

## Discussion

This study aimed to examine the potential contribution of distress and GRC to the likelihood of paying for sex, while considering attitudes toward paying for sex as a mediating factor. In addition, by delving into the mediating role of attitudes toward paying for sex, this study enhances our comprehension of a more intricate relationship wherein distress and GRC ultimately contribute to a heightened likelihood of sex payment.

According to our findings, attitudes toward paying for sex were associated with the likelihood of sex payment. Specifically, a belief in "Paying for sex as a legitimate behavior" is associated with a higher likelihood to pay for sex, while a belief in "Paying for sex as a deviant behavior" is linked to a lower likelihood of sex payment. In addition, attitudes toward paying for sex fully mediate the association between distress and most of the components of GRC (all but CBWFR) to paying for sex, implying that these attitudes play a significant role in the actualization of sex payment when distress and GRC are present. Thus, attitudes toward paying for sex, more than distress or GRC alone, appear to play a central role in shaping the likelihood of engaging in sex payment.

Previous research suggested that although the extent to which attitudes relate to behavior varies considerably (Ajzen, 2001), attitudes tend to have stronger explanatory power in certain contexts—particularly when they are directed toward the specific behavior being examined (Ajzen & Fishbein, 2005). The measure of attitudes toward men who pay for sex used in this study is indeed tightly related to the very act of paying for sex. It was also found that attitudes correlated with a future behavior more strongly when they were easy to recall (accessible) and stable over time (Glasman & Albarracín, 2006), factors that are likely to be present in an ideological, contentious domain such as paying for sex.

As mentioned earlier, previous research suggested that attitudes toward paying for sex and sex payment are related, with MPS tending to perceive paying for sex as a legitimate behavior while men who do not pay for sex tending to perceive it as a deviant behavior (Shilo et al., 2021). This relationship is assumed also by endeavors aimed at diminishing the occurrence of sex payment through the alteration of attitudes toward paying for sex, commonly known as “John Schools” (Gurd & O’Brien, 2013). Furthermore, the growing trend to criminalize sex payment in Western countries exemplifies a potential strategy for reducing paying for sex by altering attitudes. Reinforcing the perception of paying for sex as a form of deviance intends to foster decreased acceptance and tolerance of this behavior within the general population (Jonsson & Jakobsson, 2017).

Another consideration is the relationship between attitudes toward paying for sex, distress and GRC that emerges from our findings, with a positive correlation of distress and all components of GRC with both "Paying for sex as a legitimate behavior" and "Paying for sex as a deviant behavior" attitudes. A noteworthy observation is that attitudes toward paying for sex and GRC are influenced by similar social structures of gender and power, thus establishing their interconnectedness. Additionally, we suggest that the positive correlation of distress and GRC with both favoring and opposing attitudes toward sex payment represents the conflictual nature of gender roles and paying for sex. This conflictual nature was noted for instance in Shilo et al., (2021) study in which one-time sex payment was correlated with both attitudes toward paying for sex as legitimate and deviant behavior. Prior and Peled (2021) also note numerous conflicting notions surrounding the experiences of paying for sex and masculinity among men, such that sex payment both supports and compromises masculinity, provides both genuine and artificial intimacy for men*,* and allows men to occupy positions of power in relation to women while also rendering them inferior as exploited and disempowered consumers (Prior & Peled, 2023)*.*

Interestingly, the CBWFR component was an exception, as it independently associated with the likelihood of sex payment, without the mediation of attitudes toward paying for sex. Delving into the meaning of CBWFR reveals that it may deviate to some degree from the primary theoretical ambit of the other components of GRC. In his review, O’Neil (2008) summarizes the critique and the necessity for further inquiry regarding CBWFR, as despite being associated with adverse repercussions for men’s psychological well-being, it exhibits weak associations with masculinity assessments and may be applicable to women as well. We propose that CBWFR may not be closely linked to matters of sex, gender, and power like the other components, but rather to a general scarcity of time and restricted leisure resulting from the intensity of everyday life due to hard work. It has the potential to restrict men (and women) from fulfilling a physiological drive and having healthy sexual life, thus resulting in sex payment, irrespective of attitudes and even if paying for sex is perceived as deviant. Indeed, in several studies men describe paying for sex as a biological urge and as an essential need, and as preferable in terms of time, complexity, and even loyalty to spouse (Hammond & van Hooff, 2020; Huysamen & Boonzaier, 2015; Jordan, 1997).

Contrary to our initial expectations, lower levels of RABBM, not high, are associated with higher likelihood of paying for sex. A plausible explanation is that elevated levels of RABBM indicate more conservative tendencies, while higher levels denote liberalism and physical and sexual openness, enabling men to both freely interact with other men and pay for sex. This inference is grounded in prior research findings which illustrate that sexual liberals tend to hold more positive attitudes toward paying for sex than conservatives (Jakobsson & Kotsadam, 2011). For example, Robinson and Schwartz (2004) found that the RABBM component of GRC is specifically related to traditional attitudes toward the rights and roles of women. A study by Zamarripa et al. (2003) proposed that RABBM may indicate generalized restriction of affection and an overall fear of intimacy among men, with both other men and women, which may also indicate conservatism and explain reluctance to be involved in casual sexual encounters. The results concerning the distinctiveness of RABBM in relation to the other components of GRC suggest that certain circumstances can result in variations in GRC components and call for undertaking additional separate investigations on this component.

The findings also highlight religiosity as an important socio-demographic variable, as lower level of religiosity are associated with a higher likelihood of sex payment. This aligns with the widely held perception that the act of paying for sex is incompatible with religious proscriptions on various sexual behaviors (Neves, 2022), a notion that has been supported by previous research. Jakobsson and Kotsadam (2011) found a link between religiosity and attitudes toward paying for sex, with religious individuals perceiving it as morally wrong, and religiosity as being associated with an increased likelihood of supporting the criminalization of paying and getting paid for sex. Peled et al. (2020) too found that higher levels of religiosity are related to a stronger support in criminalization rather than regulation, and to a greater tendency to perceive paying for sex as a deviancy and women who are paid for sex as deviant. Further, in terms of actually paying for sex, they found that individuals who pay for sex tend to identify as secular.

### Preliminary Reflections on Support-Oriented Approaches

Although the current findings are preliminary and warrant further research, including with more diverse samples and qualitatively rich data, they offer important initial insights. To date, only a limited number of studies have examined the psychological underpinnings of sex payment through quantitative methodologies. This study is among the first to explore the associations between gender role conflict, psychological distress, and the likelihood of paying for sex among heterosexual men. Thus, the findings underscore potential psychological dynamics that may characterize some men who engage in sex payment.

Our findings align with the proposition that attitudes are a central and essential focus for intervention in endeavors aimed at influencing paying for sex among men. According to these view, targeting public attitudes and societal norms can exert a substantial influence on behavior, especially within societies characterized by collectivist values (Ybarra & Trafimow, 1998), such as Israel. Notably, an increase in Israeli public attitudes supporting criminalization of sex payment was observed in years of an intense public campaign (Peled et al., 2020) which depicted paying for sex as deviant and abusive (Prior, 2022).

Although attitudes toward paying for sex may hold considerable significance, the contribution of distress and GRC of men to their well-being and sex payment experiences cannot be underestimated. To solely focus on attitudes for intervention may prove to be a superficial solution instead of thoroughly examining the underlying causes of sex payment. Our findings indicate the significance of targeting men’s GRC in the work of helping professionals while also raising awareness of the potential detriments imposed by sexist gender roles on society at large (O’Neil, 2008).

In line with efforts to promote gender equity and to integrate psychological perspectives on masculinities and femininities (O’Neil, 2008), this study seeks to advance understanding of how gendered psychological patterns shape behavior and influence the welfare of individuals in society. Our findings emphasize the interconnection between masculine role socialization and its potential impact on both men and women in the context of paying for sex.

Our findings also emphasize the potential importance of addressing interpersonal distress related to intimate relationships in endeavors to diminish men’s sex payment, or to support to MPS who seek help, particularly in the context of GRC. Cole and Ingram (2020) suggest that the infringement upon masculine norms and the desire to uphold one’s masculinity result in a decrease in men’s willingness to seek help when experiencing psychological distress. They argue that mental health practitioners should establish outreach initiatives and develop educational resources for men, to normalize the occurrence of psychological distress and promote the utilization of psychological help-seeking. These recommendations may hold particular relevance for MPS.

These insights may contribute to emerging discussions about the types of psychosocial responses that could be relevant in contexts where sex payment is regarded as a societal concern. This is especially pertinent in countries that have adopted "end-demand" legislation, such as Israel, France, Sweden, Ireland, Norway, Northern Ireland, Iceland, and Canada. Moreover, international health and human rights organizations have emphasized the importance of promoting the mental health and well-being of all individuals involved in the sex trade, including clients. In this context, findings such as those presented here may offer a basis for tailoring non-stigmatizing, reflective, and supportive practices.

### Limitations

This study has several limitations. As a cross sectional study, no causal conclusions can be drawn. Furthermore, the generalizability of the findings to other populations and countries is limited due to the significant relevance of cultural context in this subject matter. Prior research has established that the socio-cultural context must be taken into account when examining and interpreting paying for sex related issues (Prior & Peled, 2021). Israel is characterized by pronounced collectivist values and collective norms (Yeshua-Katz & Efrat-Treister, 2021), particularly for behaviors with strong social relevance like paying for sex. Israeli collectivism can account for our findings regarding the crucial role that attitudes toward paying for sex play in the enactment of sex payment. The distinct socio-cultural context, along with its distinctive connection to paying for sex, may restrict the generalizability of our findings to other societies and warrants further exploration. The results may further vary for countries with distinct legislative policies. For example, the level of distress experienced by MPS may be considerably less significant in states where sex payment is legal or regulated.

The issue of GRC is also likely to be influenced by the local culture. For instance, CBWFR may differ in societies or countries such as Israel, where individuals lead very busy lives (OECD, 2020), potentially resulting in higher rates of sex payment compared to countries with a lower level of such conflict (e.g., fewer working hours, fewer children, less involvement of the extended family). This warrants a cross-country comparative study based on comprehensive data. Another important consideration is that the participants in this study were exclusively heterosexual men. Future studies should explore the correlations among non-heterosexual men as well. In addition, it is important to further explore the study’s findings through qualitative research, which could provide deeper insight and meaning regarding men’s attitudes, experiences of gender role conflict, and psychological distress in the context of paying for sex.

Despite these limitations, this study makes a significant and valuable contribution to the body of empirical findings regarding the motivations that underlie the phenomenon of paying for sex among men. It offers a broad and encompassing perspective that considers the influence of restrictive social norms experienced by MPS, thus adding to the existing knowledge base and expanding our understanding of sex payment.

## Data Availability

Data and questionnaire are available from the authors on demand.

## Footnotes

1 Since the Hebrew version of the measure was designed to assess public attitudes toward PS, we used the common and comprehensible Hebrew terminology for PS, which best translate as “prostitute” and “prostitution”.
